# Special Issue “Selected Papers from the 8th Asia-Pacific NMR (APNMR) Symposium: Recent Advances in NMR Spectroscopy”

**DOI:** 10.3390/ijms21124419

**Published:** 2020-06-22

**Authors:** Surajit Bhattacharjya, Daiwen Yang, Ho Sup Yoon

**Affiliations:** 1 School of Biological Sciences, 60 Nanyang Drive, Nanyang Technological University, Singapore 637551, Singapore; 2 Department of Biological Sciences, National University of Singapore, 16 Science Drive 4, Singapore 117558, Singapore

Asia-Pacific NMR (APNMR) has been an important scientific event in the region, engaging a large number of NMR scientists from academia and industries. The 8th APNMR symposium was held at the Nanyang Technological University, School of Biological Sciences, Singapore, from 3–6 July 2019. The conference had witnessed the participation of eminent scientists across the globe. The conference has covered a wide range of topics pertinent to the application and development of NMR spectroscopy. Topics included biomolecular structure and function, biomolecular dynamics, computation NMR, drug discovery, metabolomics, NMR methods, solid state NMR, natural products, and and magnetic resonance imaging (MRI) and MR spectroscopy (MRS). 

This special issue in the *International Journal of Molecular Sciences* publishes 12 invited articles selected from the conference. These articles showcase recent advancements in the broader field of NMR. Fujiwara and coworkers work on emerging cellular NMR spectroscopy that has implications in elucidating structures and dynamics in native cellular environments [1]. These authors investigate contrast agents Gd^3+^ and Gd-DOTA (gadolinium-1,4,7,10-tetraazacyclododecane-1,4,7,10-tetraacete) probing molecules in *E. coli* cells in situ [1]. Broker, Tompa, and coworkers construct melting diagrams, using a wide line NMR method, of intrinsically disordered pathogenic protein alpha-synuclein [2]. Alpha-synuclein is known to form toxic amyloid aggregates that are believed to be the leading cause of neurodegenerative Parkinson’s disease. Very little is known about how hydration influences amyloid formation. In this work, the authors observed that the mobility of the hydration layer is correlated with the extent of assembly of alpha-synuclein and its disease-causing mutant [2]. Yamada et al. develop an NMR informatics tool that can successfully reduce the noise level from a mixture of small and large molecule NMR spectra [3]. The signal deconvolution method demonstrated up to a 10-fold increase of signal-to-noise ratio and is applicable to diffusion-edited NMR spectra, permitting separation of signals from large and small molecules based on the transverse relaxation time [3]. Lu’s group analyzes tooth enamel protein amelogenin by SS-NMR, AFM, and XRD methods [4]. Their works demonstrated that the amelogenin could assume amyloid-like β-sheet structures at physiological solution conditions. Furthermore, the group describes the role of the protein in the hydroxyapatite formation of teeth [4]. The Cheng group makes a significant step forward in understanding the mechanism of action of antimicrobial peptide P113 interactions with a live fungal strain of Candida [5]. Yangmee Kim and coworkers report an NMR-derived 3D structure of an extremely thermostable acyl carrier protein from bacteria *T. maritima* [6]. The structural analysis revealed a potential mechanism for the thermostability of the protein. Weontae Lee and coworkers determine the atomic resolution structure of protein complex SNF5/BAF155, involved in chromatin remodeling [7]. The structural data has gleaned molecular insights into the tumorigenesis. Chen and Huang work on the structural characterization of mushashi-1, an intrinsically disordered protein critical for RNA granule formation [8]. The NMR study uncovered the potential role of poly-Ala regions of mushashi-1 in the assembly process. Li et al. investigate the NMR structure of CCL5 chemokine and its mutant [9]. The work underpinned the role of a tri-peptide motif sequence, F-A-Y, in forming oligomeric states of CCL5, which may be involved in the immune response. In a review article, Joon-Hwa Lee and colleagues discuss NMR structures and dynamics of various forms of the noncanonical DNA and their interactions with cognate binding proteins [10]. The authors highlight the dynamical roles of DNA in a binding mechanism. On the other hand, Li and Kang review the structures of proteases of flavivirus [11]. The structural insights would be useful in developing antiviral drugs against the proteases. Last but not least, Takeuchi and coworkers, in their review article, discuss functional dynamics, elucidated by NMR, of essential proteins, e.g., GPCR, ion channels, and GTPases [12]. The authors point out that functional dynamical characteristics may be utilized for the development of novel drug candidates. The editorial team gratefully acknowledges all the authors and the reviewers and express sincere appreciation for their significant contributions to the special issue. 
